# VIEWS ABOUT LIFE AFTER LIFE REVIEW: FROM LIFE REVIEW LEADERS’ PERSPECTIVES IN TAIWAN

**DOI:** 10.1093/geroni/igae098.2857

**Published:** 2024-12-31

**Authors:** Yi-Hsiu Lin, Tsuann Kuo

**Affiliations:** Hsiang Shang Culture and Education Foundation, Taichung City, Taichung, Taiwan (Republic of China); Chung Shan Medical University, Taichung, Taichung, Taiwan (Republic of China)

## Abstract

Guided Autobiography by Birren and Deutchman (1991) was developed and implemented as an evidence-based Life Review Program in Taiwan for over ten years. Using life course perspective, older adults were exposed to weekly topic and guided to reflect their life events. The purpose of this paper was to evaluate the impact on views about life between older adults and their program leaders after a ten-week session. This study used a mixed-method methodology to evaluate the effects of life review program on the leaders’ themselves and the older adults they served. A survey was conducted to ask leaders about the changes in older adults’ well-being, followed by qualitative interviews. Over 80 Life Review Leaders participated in this study with 87.32% were female and over 84.51% with at least a college degree. This program helped older adults communicate with family members and strengthen family bonding as well as having the courage to overcome negative events in life. The participants shared many episodes and observations that they saw older adults used each session to reflect the up and down sides of life. Older adults were able to integrate their past and forsee positive to the future life. In condusion, the structured Life Review Program helps older adults reduced depression and isolation, increased confidence and better views about self. Both clinical and policy implications can be generated to help older adults live life and face the future more positively.

